# Health system foundations for Sláintecare implementation in 2020 and beyond – co-producing a Sláintecare Living Implementation Framework with Evaluation: Learning from the Irish health system’s response to COVID-19. A mixed-methods study protocol

**DOI:** 10.12688/hrbopenres.13150.1

**Published:** 2020-09-30

**Authors:** Sara Burke, Steve Thomas, Malgorzata Stach, Paul Kavanagh, Laura Magahy, Bridget Johnston, Sarah Barry

**Affiliations:** 1 Centre for Health Policy and Management, School of Medicine, Trinity College Dublin, Trinity, Dublin 2, Ireland; 2 Health Intelligence, Strategic Planning and Transformation, 4th Floor, Jervis House, Jervis Street, Dublin 1, D01 W596, Ireland; 3 Sláintecare Programme Implementation Office, Department of Health, Department of Health Block 1 Miesian Plaza, 50-58 Lower Baggot St Dublin 2, D02XWI4, Ireland

**Keywords:** Health reform, health system, health policy, Sláintecare, COVID-19, implementation, Ireland

## Abstract

All over the world, health systems are responding to the major shock of the COVID-19 pandemic. The virus is causing urgent and fast-paced change in the delivery of health and social care as well as highlighting pre-existing deficiencies and inequalities in the health system and broader society. In Ireland, COVID-19 is occurring during the second full year of Sláintecare’s implementation – Ireland’s 10-year plan for health reform to deliver universal access to timely, integrated care.

This research will coproduce a Living Implementation Framework with Evaluation (LIFE) linking evidence, policy and practice that feeds into real-world Sláintecare implementation. In partnership with senior leadership in the Sláintecare Programme Implementation Office, the Department of Health and the HSE, the researchers will scope, document, measure and analyse the Sláintecare relevant COVID-19 responses using qualitative and quantitative methods.

The LIFE will initially take the form of a live spreadsheet which contains the COVID-19 responses most relevant to Sláintecare. For each response, 3-4 indicators will be collected which enables monitoring overtime. The spreadsheet will be accompanied by a series of rapid reviews, narrative descriptions of multiple case studies, research papers, stakeholder engagement and formative feedback. These collectively make up the ‘LIFE’, informing dialogue with the project partners, which is happening in real time (living), influencing health policy and system decision-making and implementation as the project progresses. The LIFE will inform health system reform in Ireland in the months and years after the emergence of COVID-19 as well as contributing to international health systems and policy research.

## Introduction

COVID-19 is a major shock to health systems all over the world, including the Irish health system ( WHO, 2020a) ( Government of Ireland, 2020). The arrival of the virus resulted in immediate and fast-paced change in the delivery of health and social care as well as highlighting pre-existing deficiencies and inequalities in the health system and broader society ( Burke *et al.*, 2020a; Department of Health, 2020; Murray, 2020). In Ireland, COVID-19 is occurring during the second full year of the implementation of Sláintecare – Ireland’s ten-year plan for health reform to deliver universal access to timely, integrated care ( Department of Health, 2018; Department of Health, 2019; Houses of the Oireachtas Committee on the Future of Healthcare, 2017).

‘Health system foundations for Sláintecare implementation in 2020 and beyond – coproducing a Sláintecare Living Implementation Framework with Evaluation: Learning from the Irish health system response to COVID-19’ is funded by the Health Research Board (HRB) Applied Partnership Award (APA). Dr Sara Burke is the Principle Investigator (PI) and Laura Magahy, Executive Director of the Sláintecare Programme Implementation Office in the Department of Health, is the lead knowledge user, with senior partners from the health department, the Health Service Executive (HSE), Trinity and the European Observatory on Health Systems’ and Policies.

The original aim of the project in 2019 was to utilise the development and implementation of new health regions to devise a Regional Integrated Care Area (RICA) Living Implementation Framework with Evaluation (LIFE) and this would act as a mechanism to inform overall Sláintecare implementation. With the arrival of COVID-19, in conjunction with research partners, the focus of the research project changed from learning from the regions to learning from the COVID-19 health system response. The intention is to link evidence, policy and practice, co-producing research that feeds into real-world Sláintecare implementation. Co-design and partnership approaches are inherent to this research so that the project team and health system is continuously learning and refining as the research progresses ( Health Research Board, 2019; Slattery *et al.*, 2020).

The applied approaches of health systems and policy analysis ( Buse *et al.*, 2007), ( Walt & Gilson, 1994), along with such concepts as preparedness, strengthening and reform ( Atun, 2012; WHO, 2020a; World Health, 2000; WHO, 2012), integrated care ( Atun *et al.*, 2009; Valentijn *et al.*, 2013) innovation and policy implementation inform this research ( Howlett, 2018; Nolte, 2018; Nilsen, 2015). This research will also draw on relevant literature on resilience and how health systems can cope and utilise major shocks to initiate health reform ( Thomas *et al.*, 2020; Thomas *et al.*, 2013). While there is a growing literature on health systems’ responsiveness and resilience to epidemics and pandemics, there is less research on how to use the learning from health systems’ response to a pandemic to inform health reform and the delivery of universal healthcare ( Forman *et al.*, 2020). Internationally, the UN, OECD and WHO are calling for stronger political leadership to ‘build back better’, to re-invest in sustainable and resilient health systems and deliver universal healthcare ( Clark & Gruending, 2020). This research will gather and utilise learning from the Irish health system COVID-19 response as a foundation for the broader implementation of the Sláintecare reforms.

## Study aim and objectives

This research aims to learn from the rapid, and in some instances transformative, Irish health system COVID-19 response, to assess and interpret relevance to Sláintecare’s implementation by co-producing a Sláintecare Living Implementation Framework with Evaluation (LIFE). The LIFE will scope, document, measure and analyse the relevant COVID-19 responses. This will inform Sláintecare implementation by learning with the project partners - the HSE, the Sláintecare Programme Implementation Office and the Department of Health – co-producing evidence in real-time as the project progresses.

The LIFE will initially take the form of a live spreadsheet which contains the COVID-19 responses most relevant to Sláintecare. For each response, 3–4 indicators will be collected which enables monitoring overtime. The indicators can be presented on a dashboard, accessible to health service planners, staff and users providing transparency and close to real-time information on key aspects of service provision and system reform. The spreadsheet will be accompanied by a series of rapid reviews, narrative descriptions of multiple case studies, research papers, stakeholder engagement and formative feedback. These collectively make up the ‘LIFE’, informing discussion and dialogue with the project partners, which is happening in real time (living), a formative evaluation, influencing decision-making and implementation as the research progresses.

The research will assess the Irish health system COVID-19 responses that potentially accelerate or inhibit the implementation of Sláintecare during 2020 and beyond. This will include

1. changes in entitlement to care, the experience of the service user, the reorganisation of care and
2. the way in which system change decisions were made and implemented.

The Sláintecare LIFE contributes to national and international health systems research by collecting, documenting and analysing evidence in conjunction with partners operating at the most senior level. 

The three research objectives align with three work packages outlined in the methods section:

1. **Scope the Irish COVID-19 health policy and health system response and assess if and how they are relevant to Sláintecare’s implementation in 2020 and beyond**.

The first work stream will scope the national and international response to COVID-19 and combine this with relevant theory, evidence and experience to provide the foundations to co-produce a Sláintecare LIFE.

  I. Identify, document and measure the COVID-19 health system response as most relevant to Sláintecare through coproducing a long list of COVID-19 responses and then a shortlist of Sláintecare care relevant COVID-19 responses. Identify and collect 3–4 indicators for each short-listed response assessing whether they move us closer to or further away from aims of Sláintecare – universal access to timely, integrated, quality care, better prevention, primary & community care, delivered regionally.
  II. Assess health system readiness in the current context for next steps in Sláintecare implementation specifically focussing on
    i. Learning from previous Irish health system reorganisation/reform
    ii. Delivering integrated community healthcare in the context of a crisis
    iii. Resource allocation based on population health profiling.
  III. Identify learnings from the COVID-19 response in other countries relevant to Sláintecare implementation.

2. **Identify and carry out three case studies on specific aspects of the COVID-19 response which inform/accelerate/inhibit Sláintecare’s implementation**. The second work stream will gain a deep understanding of the implementation mechanisms driving the change in order to inform Sláintecare’s implementation.
  I. Choose three in-depth cases with project partners from the shortlist of Sláintecare relevant COVID-19 health system responses outlined above. Cognisant of Sláintecare’s systems’ approach, these cases may be clusters of initiatives or enablers of health system responses.
  II. Write up the cases selected as in-depth descriptions and analyses as well identifying and collecting indicators.
  III. Situate the cases in the existing national and international literature.

3. **Objective three. Co-produce Sláintecare 2020 (COVID-19) living implementation framework with evaluation (LIFE).** This work stream links the outputs from work streams 1 & 2, clarifying how the system responds to change, creating knowledge useful for ongoing implementation and scalability.
  I. Document the implementation processes of Sláintecare relevant COVID-19 responses and identify positive implementation mechanisms i.e. what’s working to enable system change and achieve Sláintecare objectives
  II. Co-produce an early stage LIFE. Drawing on data gathered in work streams 1 & 2, the LIFE facilitates dialogue between researchers, partners and a wider audience, thereby linking evidence, policy and practice, creating knowledge which informs decision making and the implementation of Sláintecare.
  III. Document the research process and identify feedback loops as enablers of implementation (for system-learning & spread/scalability).

**Study design:** This research will use a health policy and systems research approach to co-produce a Sláintecare Living Implementation Framework with Evaluation (LIFE) with partners and key stakeholders operating in an iterative, participatory manner ( Graham & Tetroe, 2007; Sibbald *et al.*, 2014). The aim of the LIFE is to co-produce evidence from the Irish COVID-19 health system response to inform Sláintecare’s implementation and broader learning on the formulation, impact, and implementation of health policies as well as how to optimise the functioning of health systems. Quantitative and qualitative methods, rapid reviews as well as co-design and co-production techniques will be used to develop an evidence base which generates dialogue and informs policy choices and real-time Sláintecare implementation ( Chalmers, 2005). The design and approach of this research is applied ensuring the research feeds directly into Sláintecare’s priorities, implementation and health system reform in 2020 and beyond. 

Miles & Huberman (1994) affirm that a framework ‘lays out the key factors, constructs, or variables, and presumes relationships among them’ ( Miles & Huberman, 1994). The intent of this framework is to develop understanding rather than simple explanation, to move beyond identification of causal relationships towards more realist-type scoping, mapping and evaluation that can deliver on policy and practice goals ( Pawson *et al.*, 2005). The framework development process takes account of the social and complex reality of policy implementation and large-scale health system change ( Nolte, 2018) and is informed by a ‘design-orientation’ that can address system fragmentation to increase potential for application ( Tranfield *et al.*, 2003). For this research, the framework, which takes the form of the LIFE, will be used as a tool to trigger dialogue and engagement with project partners facilitating formative evaluation as a key component of this research project ( Chalmers, 2005).

**Figure 1.**
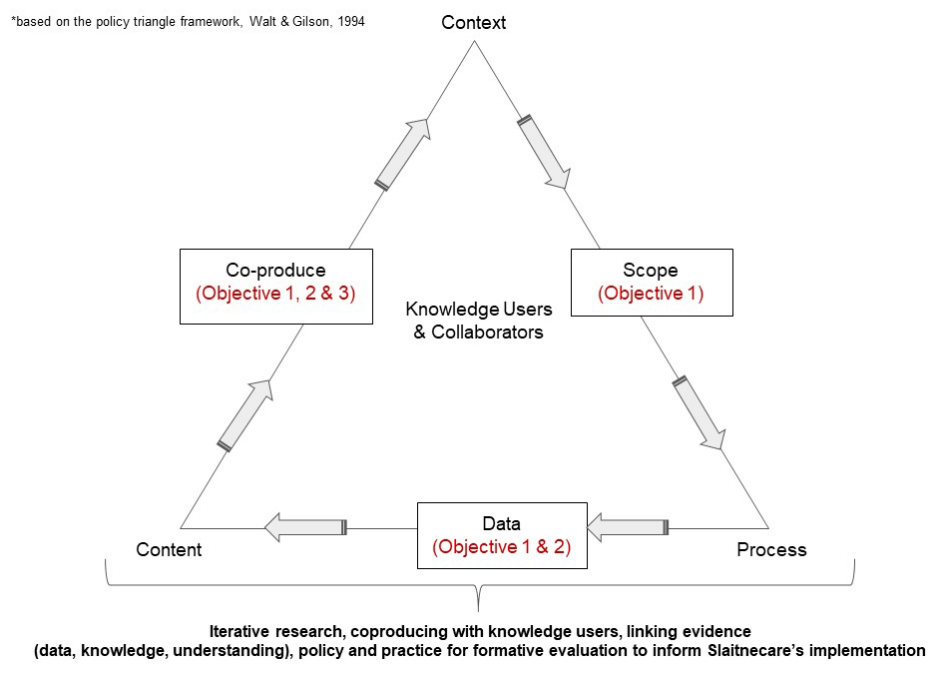
Proposed research design conceptual framework.

Co-production is at the heart of the development of the LIFE that supports the ongoing design and implementation of Sláintecare reforms through dialogue and formative evaluation with partners and relevant stakeholders ( Rycroft-Malone *et al.*, 2013; Slattery *et al.*, 2020). Close collaboration between academics, policy makers and implementers ensures better use of evidence in the policy and reform process ( Rycroft-Malone *et al.*, 2013; Rycroft-Malone, 2013). A research design framework is detailed in Figure 1, reflecting research objectives and work streams. The following methods facilitate the emergent, living, responsive, partnership nature of the research: 

## Methods

**1. Work stream 1: Scope the Irish COVID-19 health policy and health system response and assess if and how they contribute to Sláintecare’s implementation in 2020 and beyond**

1.1. ***Identify, document and measure the COVID-19 health system response most relevant to Sláintecare and assess whether these COVID-19 responses move us closer or further away from aims of Sláintecare*.**

Co-produce with project partners a long list of all Irish COVID-19 health system responses in excel. This list is being developed by the Trinity research team and project partners from documentation in the public domain as well as work we are currently doing for the Irish section of the WHO’s COVID-19 Health System Response Monitor ( WHO, 2020b). This will be supplemented and crosschecked by information submitted by project partners, who are the lead organisations directly responsible for managing and delivering the Irish COVID-19 health system response.

A shortlisting process will be co-designed with project partners. The aim of the shortlisting is to

- Agree a list of COVID-19 responses most relevant to Sláintecare
- Short list potential case studies
- Provide data for the initial Sláintecare LIFE.

Each shortlisted health system response will be written up as a narrative description. For each shortlisted response, 3–4 indicators will be selected to allow progress to be monitored over time, facilitating an assessment of which COVID-19 responses enhance or inhibit Sláintecare’s implementation.

The indicators selected for this work will be informed by Health System Performance Assessment (HSPAs), currently used by the WHO, OECD and in Europe as a mechanism to understand how health systems work and how to carry out actions to improve them ( OECD, 2020; World Health, 2000). HSPAs offer transparency that is essential to improve health systems’ accountability and functioning, allowing macro-level cross-country comparisons.

Indicators for this project will also be informed by previous work of the research team and other Irish work ( Burke *et al.*, 2014; Harnett *et al.*, 2020; National Clinical and Integrated Care Programmes, 2018) and work currently underway in the Irish Department of Health developing indicators of Performance Accountability for the Irish Health System ( Health Systems Research Unit, 2020).

The starting point will be selecting indicators from the short list of Sláintecare relevant COVID-19 responses at a national level. Where possible pre-COVID-19 data will be used to facilitate time-series analysis. This will be a resource for all partners and provides a basis for formative evaluation. Developing indicators to monitor progress towards universal healthcare in high-income countries is difficult ( Bergen *et al.*, 2019). This aspect of the research will assist in such development in an Irish context as well as contributing to international knowledge in this under-developed area.

**1.2. *Assess health system readiness for next steps in Sláintecare implementation specifically focussing on learning from previous Irish health system reorganisation, delivering integrated care in the community in the context of a crisis and resource allocation based on population health profiling*.**

Prior to the arrival of COVID-19, one of the main areas for focus of the implementation of Sláintecare was the design and implementation of the new Regional Health Areas ( Health Service Executive, 2019; Department of Health, 2019). These developments were paused in early 2020 as the entire health system focus was on coping with COVID-19. Other pre-COVID-19 implementation priorities included; the reorientation of the health system so that much more care is provided outside of hospital in the community as close as possible to people’s homes; and how to allocate resources on the basis of population health need ( Department of Health, 2019).

In order to assess health system readiness for the next stages of Sláintecare’s implementation, the narrative descriptions and indicators developed above will be supplemented with in-depth research including rapid reviews on:

  1.2.1. ***Understanding Service Reorganisation in the Irish Health & Social Care System 1998 to 2020:*** This research will examine how we can learn from previous health system reorganisations in Ireland and internationally. There are three components of this research: 1) analysis of key Irish policy and strategy documents relevant to reorganisation; 2) interviews with elite informants; 3) a rapid review of peer-reviewed literature on reorganisation in health and social care systems. This mixed methods research will generate a rich and useful ‘narrative of change’ highlighting the phases, processes, implementation strengths and weaknesses reorganisation in the Irish health and social care system from 1998 to 2020 situating the findings in the context of COVID-19 health system response.
  1.2.2. ***Delivering integrated care in the community in the context of a crisis:*** The delivery of universal access to integrated care was the core vision of the 2017 Sláintecare Oireachtas Committee report ( Houses of the Oireachtas Committee on the Future of Healthcare, 2017). The 2019 Sláintecare Action Plan ‘focuses on providing the right care, in the right place, at the right time... Programmes will design integrated services to provide care and support at, or near, home where appropriate and to ensure hospital stays are minimised’. In order to inform Irish work in this context, the research team will conduct a rapid review of international literature on delivering integrated care in the community in the context of a crisis.
  1.2.3. ***Resource allocation based on population health profiling*:** Sláintecare recommended that resources be allocated on the basis of population health need as a key mechanism to delivering system reform. This rapid review will inform Irish health policy by describing models of population-based resource allocation and synthesising evidence about their implementation and impact. It will appraise peer-reviewed and grey literature using streamlined systematic review methods.

These reviews combined will inform a short research brief on the readiness of the health system to reform and deliver Sláintecare in light of the COVID-19 response. Health system readiness literature is relevant as it is considered critical for successful implementation of complex interventions ( O’Neill *et al.*, 2013). Many of the readiness dimensions are common to the WHO Building Blocks taking a systems’ approach which informed the original Sláintecare report ( World Health, 2013; Burke *et al.*, 2018). This work will inform Sláintecare priorities in 2020 and beyond.

Rapid reviews are the primary method for this component due the applied nature of this project and the practicalities of time. There is little consensus on agreed definitions or methods for conducting rapid reviews ( Haby *et al.*, 2016). However, Haby *et al.* concluded that researchers should be explicit about their methods, in particular efforts to reduce the timeframe of a usual systematic review, by limiting the scope of the review, restricting the study types, limiting data extraction to key characteristics, specifying that the review should explain the implications of these limitations ( Haby *et al.*, 2016).

This research utilises Grant’s definition of a rapid review as an ‘assessment of what is already known about a policy or practice issue, by using systematic review methods to search and critically appraise existing research... whose completeness of searching determined by time constraints… where the appraisal is a time-limited formal quality assessment.. The synthesis is typically narrative and tabular and analyses the quantity of literature and overall quality/direction of effect of the literature’ ( Grant & Booth, 2009: 5).

The core principles chosen to drive the quality of the reviews are in line with the methodological approach of management and organisational science:

- Transparent – are the methods/findings/implications clearly presented and plausible?
- Inclusive – does the review reflect the complexity of the phenomenon under study?
- Explanatory – does the review explain the complex patterning (factors, causal pathways, contextual issues etc.) which constitutes the phenomenon under study?
- Heuristic – does the review highlight the implications of its findings to a given policy projection including attention to practical concerns such as feasibility and the political context? ( Denyer & Tranfield, 2009).

***1.3 Identify relevant learnings from the COVID-19 response in other countries relevant to Sláintecare implementation***

The PI and project team members are also the authors of the Irish page of the WHO, EU and European Observatory on Health Systems’ & Policy COVID-19 Health System Response Monitor ( Burke *et al.*, 2020a) and contribute to the Cambridge Core blog on the Irish Country Responses to the COVID 19 Pandemic ( Burke *et al.*, 2020b). The WHO Health System Response Monitor includes analysis of trends and key lessons across countries ( WHO Regional Office for Europe, 2020). This allows the research team to draw on existing analyses and to carry out a cross-country analysis in conjunction with members of the European Observatory on Health Systems and Policies’. As appropriate the team will utilise this international comparative work to inform aspects of work streams 1 and 2.

**2. Work stream 2. Identify and carry out three case studies on aspects of the COVID-19 response which inform/accelerate/inhibit Sláintecare implementation.**

***2.1 From the shortlist of Sláintecare relevant COVID-19 responses, the research team and partners will select three in-depth cases. Cognisant of Sláintecare’s systems’ approach, these cases may be clusters of initiatives or enablers of the health system response.***
***2.2 The cases selected will be written up as an in-depth narrative analysis as will the identification and collection of structural, process, output and outcome indicators.***
***2.3 Situate the cases in the existing national and international literature.***

Three case studies will be carried out using documentary analysis and semi-structured interviews ( Yin, 2003).

*Documentary analysis:* The first stage of the data collection and analysis of the case studies will be to assemble all available documentary information relevant to the cases as well as data sources for potential indicators. Documentary analysis is used to understand the substance of documents, putting them in context, explaining their significance, and giving a summary ( Prior, 2011). The researchers will gather documents for this research largely through obtaining documentation from project partners with detail on the specific initiatives. These will include minutes of meetings, emails, project plans, terms of references of specific groups set up as part of the COVID-19 response as well as governance structures.

*Semi-structured interviews:* A weakness of the documents is that they tell the ‘official’ narrative, outlining what decisions were made not necessarily reflecting the reality of operations or actual practice ( Silverman, 2011): 96). Therefore, up to 12 interviews (3–4 per case) will be conducted with senior managers, policy makers, researchers and clinicians to supplement official documentation. Interviewees will be identified through purposive and snowball sampling. A semi-structured interview protocol will be developed in advance of the interviews drawing on key issues emerging from the documentation and other research strands in consultation with the research team and project partners. The content of the interviews will be recorded, transcribed and imported into a software package. The interview data will coded by two researchers.

The cases will be written up as briefing papers including the identification of indicators as well as initial analysis of common enablers and mechanisms emerging across the cases.

**3. Work stream 3. Co-produce a Sláintecare 2020 (COVID-19) living implementation framework with evaluation (LIFE).**

***3.1 Document the implementation processes of Sláintecare relevant COVID-19 responses and identify positive Sláintecare relevant COVID 19 implementation mechanisms***

The research team will document the implementation processes of Sláintecare relevant COVID-19 responses through documentary analysis, interviews, critical conversations and dialogue with partners as carried out for the previous work streams. We will capture the implementing story as well as identifying the critical enablers and mechanisms for change drawing on key implementation literature ( Braithwaite *et al.*, 2018; Ghate, 2016; Howlett, 2018). Existing systems fora and networks will be utilised to gain further insight into implementation mechanisms. These include the Sláintecare Advisory Council, the Sláintecare Integration Fund, the HSE board, the HSE and Department of Health management teams, health service unions’, other opportunistic webinars and events as they arise. The enablers and mechanisms for change will be fed back to project partners as part of the formative evaluation as the research progresses, informing research briefs and research articles.

***3.2 Coproduce an early stage living implementation framework with evaluation (LIFE).***

Drawing on the various components of this research, the research team and partners will co-produce a Sláintecare LIFE. The LIFE will initially take the form of a live spreadsheet of indicators of COVID-19 health system responses most relevant to Sláintecare. This potentially can be presented as an online dashboard, accessible to health service planners, staff and users providing transparency and close to real-time information on key aspects of service provision and system reform. The indicator monitoring and assessment combined with the rapid reviews, narrative descriptions of multiple case studies, research papers and stakeholder engagement as detailed above form the basis of ongoing dialogue with the project partners and make up the ‘LIFE’. Dialogue between project partners on the LIFE is a form of formative evaluation, whereby linking evidence, policy and practice is used to inform decision making in real-time, as the ‘living’ research progresses.

The term ‘evidence-informed’ is utilised here to mean using the best available data and research evidence, systematically and transparently, in the time available at each stage of the policy cycle ( Chalmers, 2005). These indicators should be ‘mainstreamed’ i.e. collected and published regularly by the Sláintecare Implementation Programme Office and the HSE during and after the lifetime of this project so that they can be monitored over time. This allows transparency. They should have an in-built review to ensure they are the best indicators, especially as new data become available which may be better indicators of impact.

***3.3 Document the research process and identify feedback loops as implementation enablers (for system-learning & spread/scalability).***

At the essence of this research is partnership working and co-production. This manifests itself in ongoing regular contact by phone, email, meetings between the research team and partners. This partnership working began nearly a year before the research was funded and although has changed due to COVID-19 it continues. For example, from April to Autumn 2020 there are regular online steering group meetings to oversee this project. The research findings are continuously fed to partners and the partners are continuously feeding into the research. As well as structured events, there are opportunistic events which will enable further engagement with senior policy makers and managers as well as frontline staff, patient, carers and community representatives.

This reflective, formative, emergent, iterative, responsive approach enables real-time continuous knowledge that provides the knowledge users (Sláintecare Programme Implementation Office and the HSE) with an important stream of data, knowledge and reflexive awareness to assist in delivering change in the midst of a pandemic. This also informs Sláintecare implementation. More broadly this will contribute to national and international health system reform through documenting and learning from the methods and approach of the Foundations’ project, paying particular attention to identifying feedback loops. 

## Results dissemination

As this is a HRB Applied Partnership Award (APA) and the nature of the applied research is co-production, the research findings are continuously fed to partners formally and informally throughout the project ( Health Research Board, 2019). There will be a range of research outputs from the research project (rapid reviews, academic journal articles, research papers and policy briefs, published indicators) which will be disseminated through the Centre website, partners’ and academic networks and journal publications. Results will be reported in line with GRADE-CERQual, COREQ and PRISMA as appropriate ( Booth, 2014; Lewin *et al.*, 2018; Moher *et al.*, 2009).

## Study status

The research is currently underway.

## Discussion

This research draws on the PI and research team’s track record of high quality, innovative Irish health systems’ and policy research. Due to the applied nature of the funding, the partners, the HSE and the Sláintecare Programme Implementation Office have been involved since the research’s inception and are continuously working in partnership with the researchers, providing access to data, coproducing the study design, findings and learning from the research as it progresses. The Foundations’ research is an exciting test case for formative, applied health system research in which the worlds of academia and senior Irish health policy makers and implementers work together to influence the delivery of Sláintecare (providing universal access to timely, quality, integrated care and improved population health and well-being) as well as contributing to international literature on health systems reform.

## Ethical approval and consent

Ethical approval was sought for a pre-COVID version of this project in November 2019 from the Centre for Health Policy/Centre for Global Health Research Ethics Committee in the School of Medicine, Trinity College Dublin and secured in January 2020 following some amendments to the original proposal (Ethics approval number - 11/2019/04E). While the focus of the research has changed, the methods have not so no further ethics is required. This protocol will be forwarded to the TCD ethics committee for their information and records. The vast majority of the secondary analysis of data in the public domain. Primary data will be obtained from the interviews, informed consent forms and participant information leaflets for any participants were approved in the ethics process. 

## Data availability

No data is associated with this article.
