# Supplementary material for: Health system foundations for Sláintecare implementation in 2020 and beyond – co-producing a Sláintecare Living Implementation Framework with Evaluation: Learning from the Irish health system’s response to COVID-19. A mixed-methods study protocol
Source: HRB Open Res. 2020 Sep 30;3:70. [Version 1] doi: 10.12688/hrbopenres.13150.1 (PMC7934093; doi:10.12688/hrbopenres.13150.1)
Supplement: Peer review history

# Peer review: Reviewer response for version 1

**DOI:** 10.21956/hrbopenres.14267.r29053

**Published:** 2021-03-04

**Authors:** Brad Wright

**Affiliations:** 1 Department of Family Medicine, University of North Carolina at Chapel Hill, Chapel Hill, NC, USA; 2 Cecil G. Sheps Center for Health Services Research, University of North Carolina at Chapel Hill, Chapel Hill, NC, USA

This manuscript presents a study protocol that will use the Irish health system's rapid-response to COVID-19 to essentially implement a rapid-cycle review that can also be used to inform the evaluation of Slaintecare implementation. The goal is a study that includes a scoping review, 3 in-depth case studies, and the co-production of the living implementation framework with evaluation. My only criticism here is that the authors refer to this as a "mixed-methods" study. In my experience and in my field of health policy and health services research, this term is generally used to describe studies that employ some mixture of both quantitative and qualitative research methodology. While the authors do use different research methods, I would classify all of them as qualitative in nature, and would not label them as "mixed-methods". Otherwise, I thought this manuscript was quite well-written and have no further critiques.

Is the study design appropriate for the research question?

Yes

Is the rationale for, and objectives of, the study clearly described?

Yes

Are sufficient details of the methods provided to allow replication by others?

Yes

Are the datasets clearly presented in a useable and accessible format?

Not applicable

Reviewer Expertise:

Health policy and health services research

I confirm that I have read this submission and believe that I have an appropriate level of expertise to confirm that it is of an acceptable scientific standard.

# Peer review: Reviewer response for version 1

**DOI:** 10.21956/hrbopenres.14267.r28236

**Published:** 2020-11-10

**Authors:** Anne Matthews

**Affiliations:** 1 School of Nursing, Psychotherapy & Community Health, Dublin City University, Dublin, Ireland

Overall this is an excellent protocol for a very welcome mixed methods study of how COVID-19 related health system responses and changes might be better understood to support the implementation of Ireland’s 10-year health strategy, Sláintecare. The shifting of the study towards COVID-19 related learning, from the original plan to study the new health regions is well explained and justified in the Introduction. The focus on COVID-19 responses (on entitlement, experience and reorganisation of care; and on the decision-making behind these changes) are well chosen. The three research objectives and associated work streams are clearly communicated with the appropriate and very ambitious mixed methods are described in great detail. The proposed research design framework effectively adapts the Walt and Gilson’s iterative policy analysis triangle, with its four factors of context, actors, content and process. The adaptation takes specific account of the proposed study’s features, for example, building in co-production. Ethical approval was obtained for pre-COVID version of this study and these changes will be notified to the relevant committee. It is a co-designed project with knowledge users from key policy and implementing organisations. The reference list is very comprehensive and is a great resource in itself. This team of researchers seem best placed to do this work and already contribute about Ireland’s health system and (even) its COVID-19 responses to relevant comparative European platforms and publications. This protocol was a pleasure to review and this publication in itself is instantly relevant for many people, including me, and the other anticipated varied study outputs will be eagerly awaited.

 Given the high quality of the protocol, especially the detail within the methods section, only minor questions arose for me, such as:

- What does the ‘Health system foundations’ phrase at the start of the title mean or add to the title, is it necessary?
- Make clearer in the abstract and paragraph 2 of Study aims and elsewhere:  what does a ' live spreadsheet' mean? 
- This project was co-designed and the LIFE is being co-produced with knowledge users who are the relevant policy-makers and implementers; was there public/ patient involvement or is this planned (if not, maybe a brief point about this could be added)?

Minor typos:

 Author list- make clear that ‘Health Intelligence’ is the Health Intelligence Unit of the HSE (as is clearer in the ‘competing interests’ section).

Page 3

Introduction

 paragraph 2

- Change Principle to Principal
- Clarify who are "senior partners from the health department" as being from/within the Department of Health or from another department?
- Change Trinity to ‘Trinity College Dublin’?
- Take out the apostrophe after Systems

Study aims and objectives

 The terms ‘the Sláintecare LIFE' and ‘the LIFE’ are used in different places throughout, maybe use one or the other for visibility as the project develops?

Methods

 Page 8, Last sentence of section 3.2 (and within the Discussion section:  the reference to 'The Foundations’ research' is not clear (this may also relate to the query about the title).

Reference list: Change Who to WHO (or spell out first/ all or none of WHO references)?

Is the study design appropriate for the research question?

Yes

Is the rationale for, and objectives of, the study clearly described?

Yes

Are sufficient details of the methods provided to allow replication by others?

Yes

Are the datasets clearly presented in a useable and accessible format?

Not applicable

Reviewer Expertise:

Health policy; Maternal, newborn and child health; Infant feeding/ Breastfeeding.

I confirm that I have read this submission and believe that I have an appropriate level of expertise to confirm that it is of an acceptable scientific standard.
